# Supplementary material for: Crosstalk between glucocorticoid and mineralocorticoid receptors boosts glucocorticoid-induced killing of multiple myeloma cells
Source: Cell Mol Life Sci. 2023 Aug 14;80(9):249. doi: 10.1007/s00018-023-04900-x (PMC10425521; doi:10.1007/s00018-023-04900-x)
Supplement: Supplementary file 1 — Supplementary file1 (PDF 3310 KB) [file 18_2023_4900_MOESM1_ESM.pdf]

Supplementary Information

Figure S1

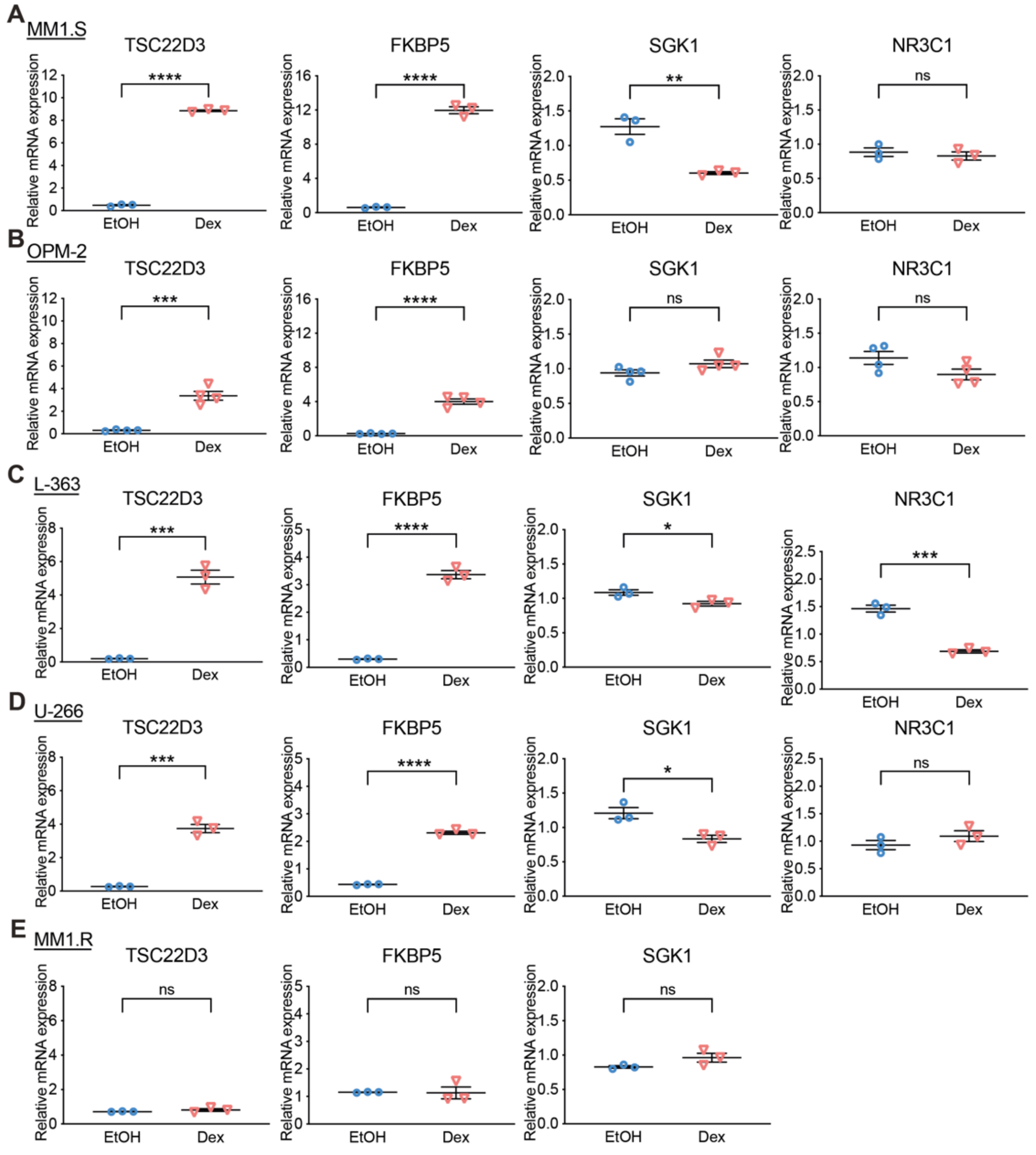

Fig. S1: Target genes of GR and MR are differentially regulated at the mRNA level between MM cell lines with varying GC responsiveness.

(**A-E**) Different myeloma cell lines, i.e. (**A**) MM1.S (N=3) (**B**) OPM-2 (N=4), (**C**) L-363 (N=3), (**D**) U-266 (N=3) and (**E**) MM1.R (N=3) cells were treated for 6h with Dex (10^{-6}M) or solvent control (EtOH). RNA was isolated and subjected to RT-qPCR analyses, hereby assaying the mRNA levels of *TSC22D3* (GILZ), *FKBP5*, *SGK1* and *NR3C1* (GR). Note that *NR3C1* is not expressed in MM1.R cells. Data analyses were performed using qBaseplus with *SDHA*, *RPL13A* and *YWHAZ* serving as reference genes. Note that the mRNA levels of the targets of interest are normalized to those of the above-mentioned reference genes (relative mRNA expression in the y-axis). The scatter plots represent the mean (solid line) +/- SEM. Statistical analyses were performed using GraphPad Prism 9, using a two-sided unpaired t-test.

Figure S2

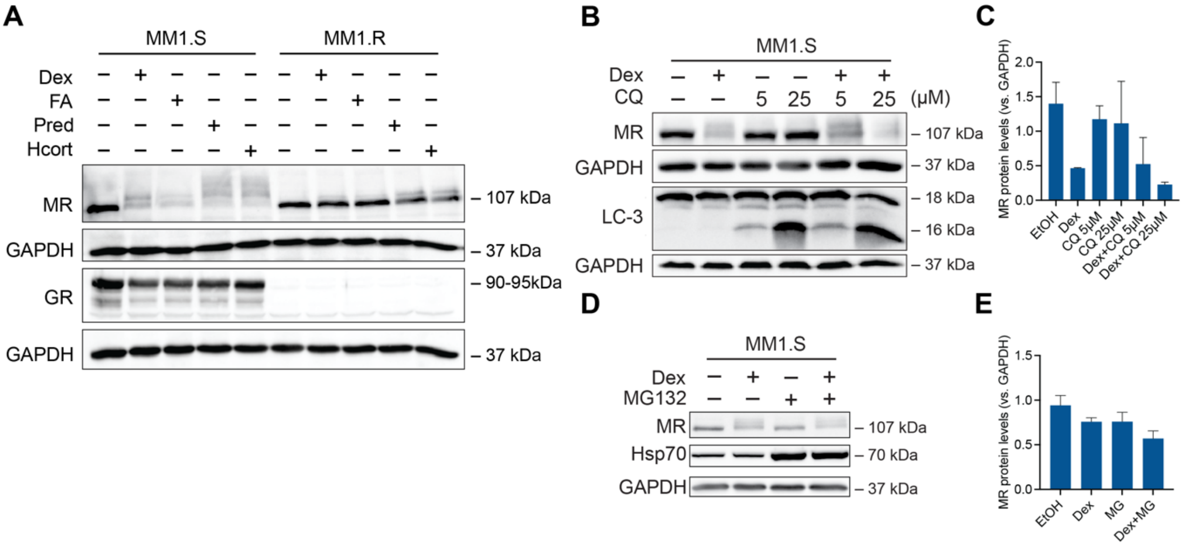

Fig. S2: MR protein levels are also downregulated by other GCs and neither proteasomal nor lysosomal degradation are involved.

(**A**) MM1.S and MM1.R cells were treated with Dex, FA, Pred, Hcort (10^{-6}M each), and analyzed via WB (N=3).

(**B-C**) MM1.S cells were treated for 24h with solvent, Dex (10^{-6}M), CQ (5.10^{-6}M or 25.10^{-6}M), a Dex/CQ combination or solvent control, followed by (**B**) WB analyses (N=2) and (**C**) band densitometric analysis.

(**D-E**) MM1.S cells were treated for 6h with Dex (10^{-6}M), MG132 (10^{-6}M), a Dex/MG132 combination or solvent control (N=3), followed by (**D**) WB analyses (N=3) and (**E**) band densitometric analysis.

Data information: (**A**, **B, D**) Protein lysates were subjected to WB analysis, determining the protein levels of GR (90-95kDa), MR (107kDa), LC-3 (16-18kDa; positive control for inhibition of lysosomal degradation) or Hsp70 (70kDa; positive control for proteasome inhibition). GAPDH (37kDa) served as a loading control. One representative image for each WB experiment is shown, with the number of biological replicates mentioned in each panel description. (**C, E**) Band densitometric analysis (bar plot) shows the normalized MR protein levels (vs. GAPDH), averaged over the biological experiments +/- SEM.

Figure S3

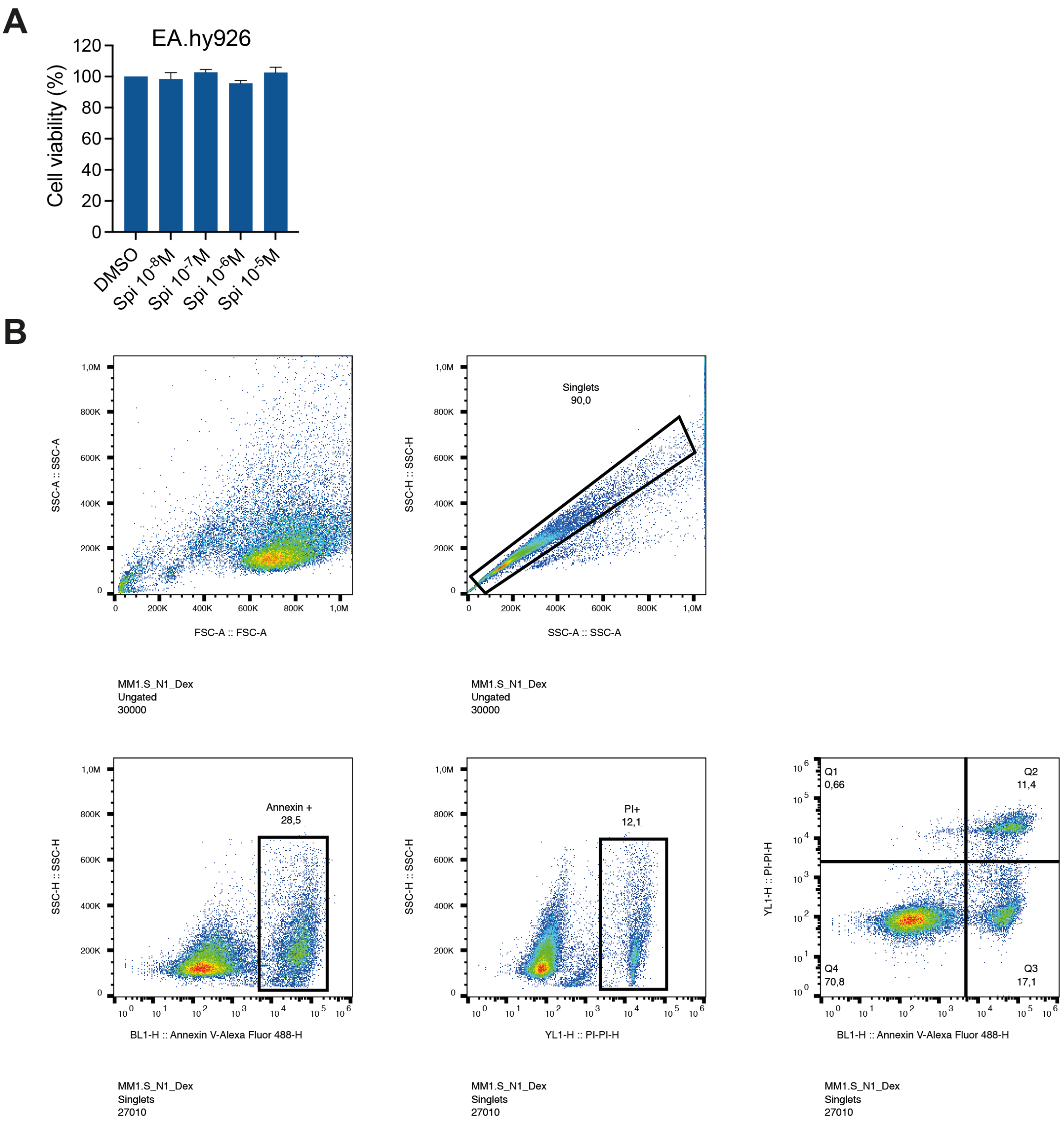

Fig.S3: EA.hy926 cell-based toxicity control for Spi treatment along with the gating strategy of the flow cytometric analyses in MM1.S cells.

(**A**) EA.hy926 cells were treated for 72h with a Spi concentration range (10^{-5}M-10^{-8}M), followed by CellTiterGlo cell viability assays (N=3). Solvent control (DMSO) was set at 100% and all other conditions were recalculated accordingly. The bar plot represents the average +/- SEM. Statistical analyses were performed using GraphPad Prism 9, using one-way ANOVA with post-hoc testing, comparing 10^{-8}M Spi with all other Spi concentrations. No significant differences were found.

(**B**) Gating strategy for Annexin V/PI flow cytometric analyses, illustrated for a Dex-treated sample.

Figure S4

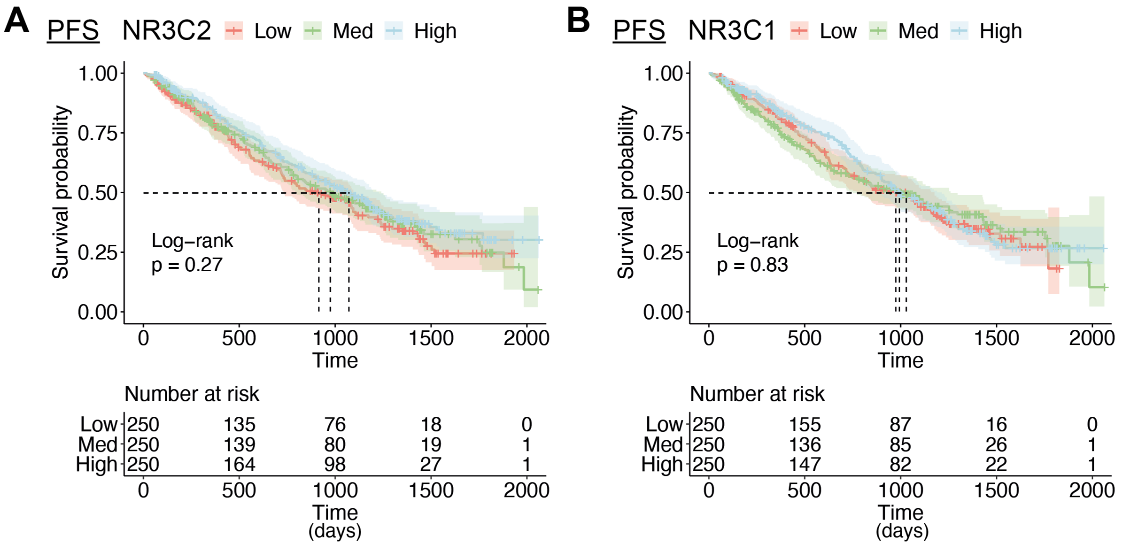

Fig. S4: Link between *NR3C2* or *NR3C1* expression levels and survival characteristics.

(**A-B**) Kaplan-Meier curves of the MMRF patient cohort, depicting the survival probability in function of progression-free survival (PFS) for low, medium or high expression of (**A**) *NR3C2* (MR) or (**B**) *NR3C1* (GR). Patients were divided in 3 groups based on their expression levels of *NR3C2* or *NR3C1*. Statistical analyses were performed in R (package survival), using a log-rank test.

Figure S5

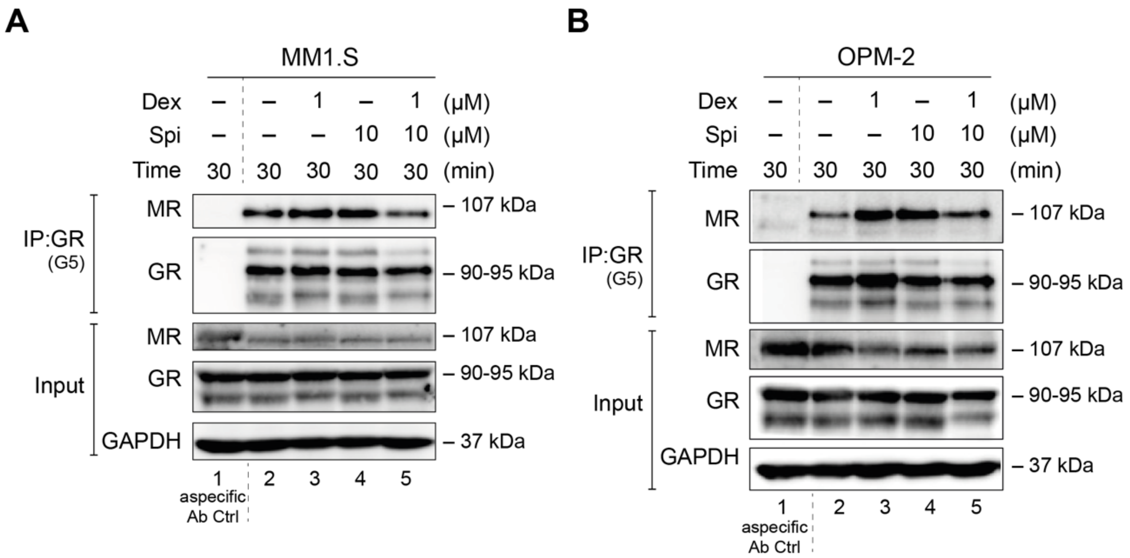

Fig. S5: Biological replicates of the co-IPs of GR with MR in MM1.S and OPM-2 cell lines.
(**A-B**) Two myeloma cell lines, i.e. (**A**) MM1.S and (**B**) OPM-2 cells, were treated with Dex (10^{-6}M), Spi (10^{-5}M), a Dex-Spi combination or solvent control for 30min. Protein lysates were prepared and subjected to endogenous IP using GR (G5) antibody. Thereafter, WB analyses were performed to determine co-IP of GR (90-95kDa) with MR (107kDa); GAPDH served as loading control for the input fraction. Lane 1 represents the non-specific antibody control. These WBs represent the second biological replicate of the co-IPs presented in Fig.5D,F.

Figure S6

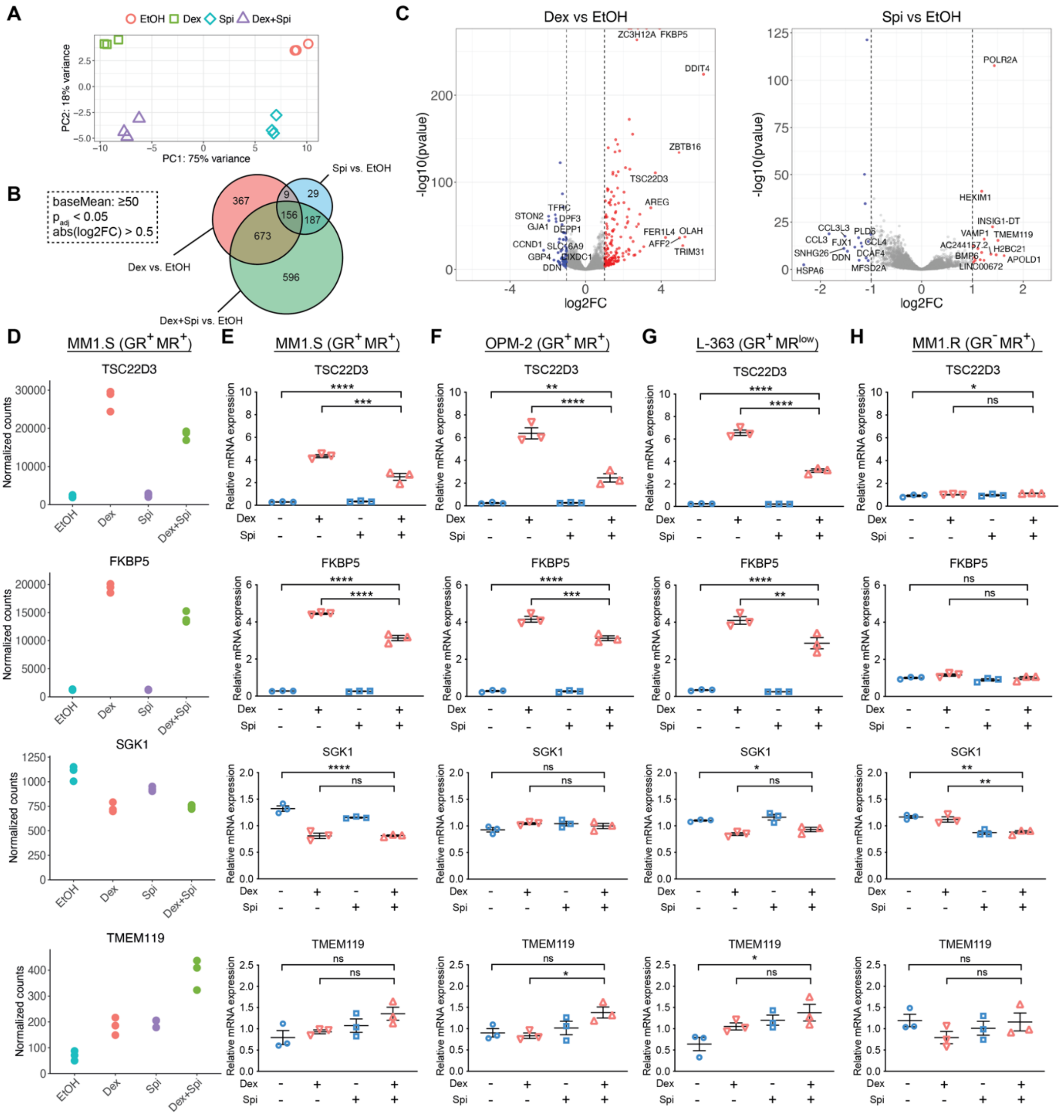

Fig. S6: Validation of the RNA-sequencing results at the mRNA level in several myeloma cell lines.

(**A-D**) MM1.S cells were treated with Dex (10^{-6}M), Spi (10^{-5}M), a Dex/Spi combination or solvent control (EtOH) for 6h, followed by RNA-seq analysis. (**A**) Principal component analysis of each condition and biological replicate of the RNA-seq experimental setup (6h treatment, N=3).

(**B**) Venn-diagram depicting the number of protein coding genes with baseMean≥50 that are significantly regulated (p_{adj}<0.05) and have an abs(log2FC)>0.5 in and between different pairwise comparisons (Dex-Spi vs EtOH; Dex vs EtOH; Spi vs EtOH).

(**C**) Volcano plots depicting the p_{adj} (log10 scale) in function of the log2FC for all genes with baseMean ≥50 for the pairwise comparison Dex vs EtOH or Spi vs EtOH. Significantly regulated genes (p_{adj}<0.05) are colored in red when log2FC>1 or blue when log2FC<-1; non-significant genes (p_{adj}>0.05) in grey. The gene names are displayed for those genes having the largest abs(log2FC) values (top 10 upregulated/downregulated).

(**D**) The normalized counts are plotted for several genes identified by RNA-sequencing in MM1.S.

(**E-H**) Several myeloma cell lines, i.e. (**E**) MM1.S, (**F**) OPM-2, (**G**) L-363 and (**H**) MM1.R cells were treated with Dex (10^{-6}M), Spi (10^{-5}M), a Dex/Spi combination or solvent control (EtOH) for 6h (all cell lines N=3). RNA isolation and RT-qPCR analyses were performed to determine the mRNA expression levels of *TSC22D3* (GILZ), *FKBP5*, *SGK1* and *TMEM119*. Data analyses were performed using qBaseplus with *SDHA*, *RPL13A* and *YWHAZ* serving as reference genes. Note that the mRNA levels of the targets of interest are normalized to those of the above-mentioned reference genes (relative mRNA expression in the y-axis). The scatter plots represent the mean +/- SEM (N=3 for all cell lines). Statistical analyses were performed using GraphPad Prism 9, using a one-way ANOVA with post-hoc testing.

Figure S7

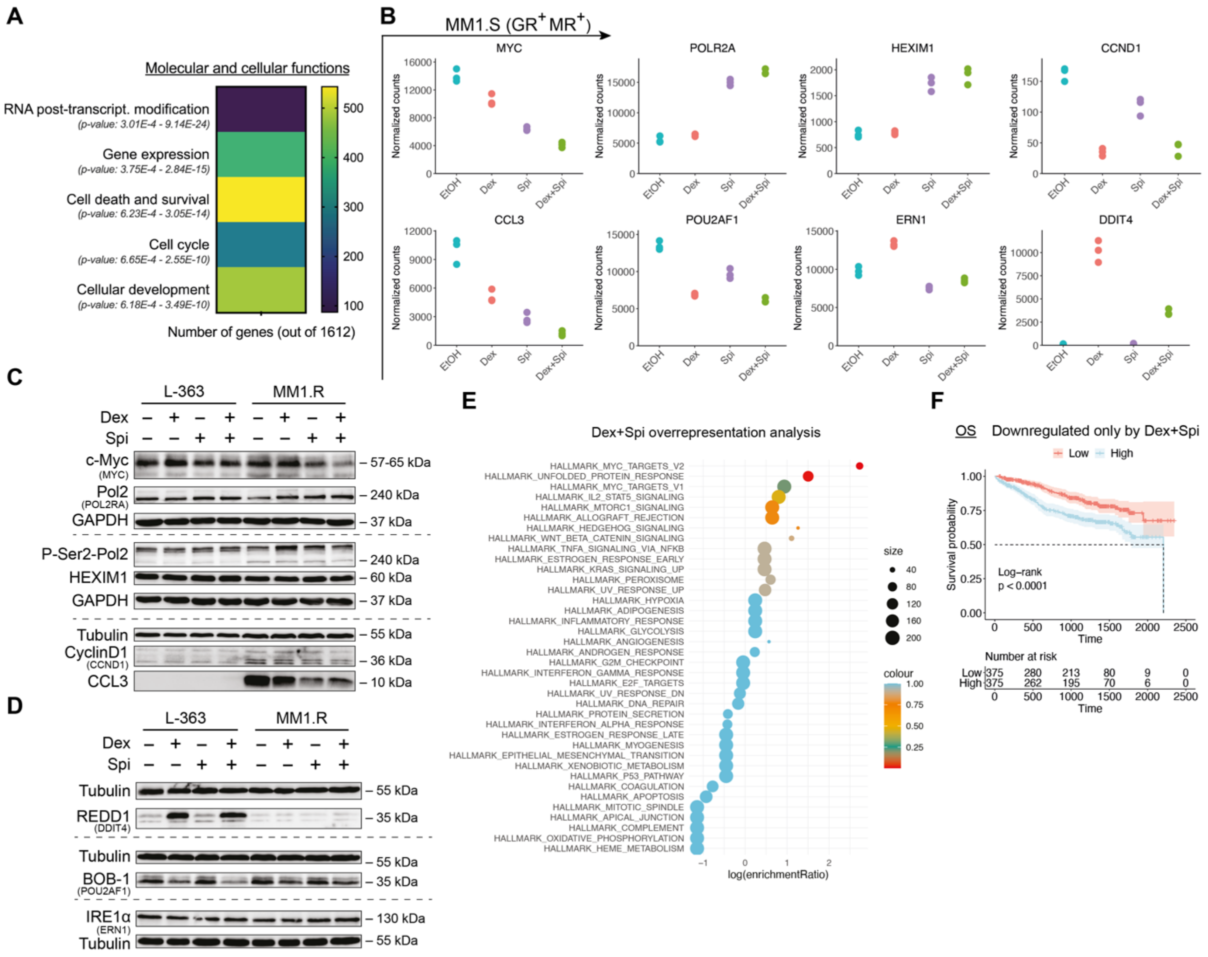

Fig. S7: Validation of the RNA-sequencing results at the protein level in several myeloma cell lines along with functional annotations.

(**A**) Molecular and cellular functions identified via core analyses in IPA using protein coding genes with baseMean≥50, p_{adj}<0.05 and abs(log2FC)≥0.5 as input for the pairwise comparisons Dex-Spi vs EtOH. The number of genes that are allocated to a certain molecular and cellular function are depicted as a heatmap and the corresponding p-value range is mentioned.

(**B**) The normalized counts are plotted for several genes identified by RNA-sequencing in MM1.S.

(**C**) L-363 and MM1.R cells were treated with Dex (10^{-6}M), Spi (10^{-5}M), a Dex/Spi combination or solvent control (EtOH) for 24h (both N=3). Protein lysates were prepared and subjected to WB analyses, hereby assessing the protein levels of c-myc (*MYC*, 57-65kDa), (P-Ser2) RNA-Pol2 (*POLR2A*, 240kDa), HEXIM (*HEXIM1*, 60kDa), cyclinD1 (*CCND1*, 36kDa) and MIP-1α (*CCL3*, 10kDa).

(**D**) L-363 and MM1.R cells were treated with Dex (10^{-6}M), Spi (10^{-5}M), a Dex/Spi combination or solvent control (EtOH) for 24h (both N=3). Protein lysates were prepared and subjected to WB analyses, hereby assessing the protein levels of REDD1(*DDIT4*, 35kDa), BOB-1 (*POU2AF1*, 35kDa) and IRE1α (*ERN1*, 110-130kDa). Tubulin (55kDa) served as a loading control.

(**E**) GSEA-based overrepresentation analysis for the genes uniquely downregulated by Dex-Spi, hereby identifying hallmarks that are enriched. Color refers to the significance level (p-value) and size to the number of enriched genes per hallmark.

(**F**) Kaplan-Meier curve of the CoMMpass patient cohort (N=750), depicting the survival probability in function of overall survival (OS) for low or high expression of genes that were uniquely downregulated by the Dex-Spi combination. Statistical analyses were performed in R (package survival), using a log-rank test.

Data information: (**C, D**) One representative image for each WB experiment is shown, with the number of biological replicates mentioned in each panel description.

Figure S8

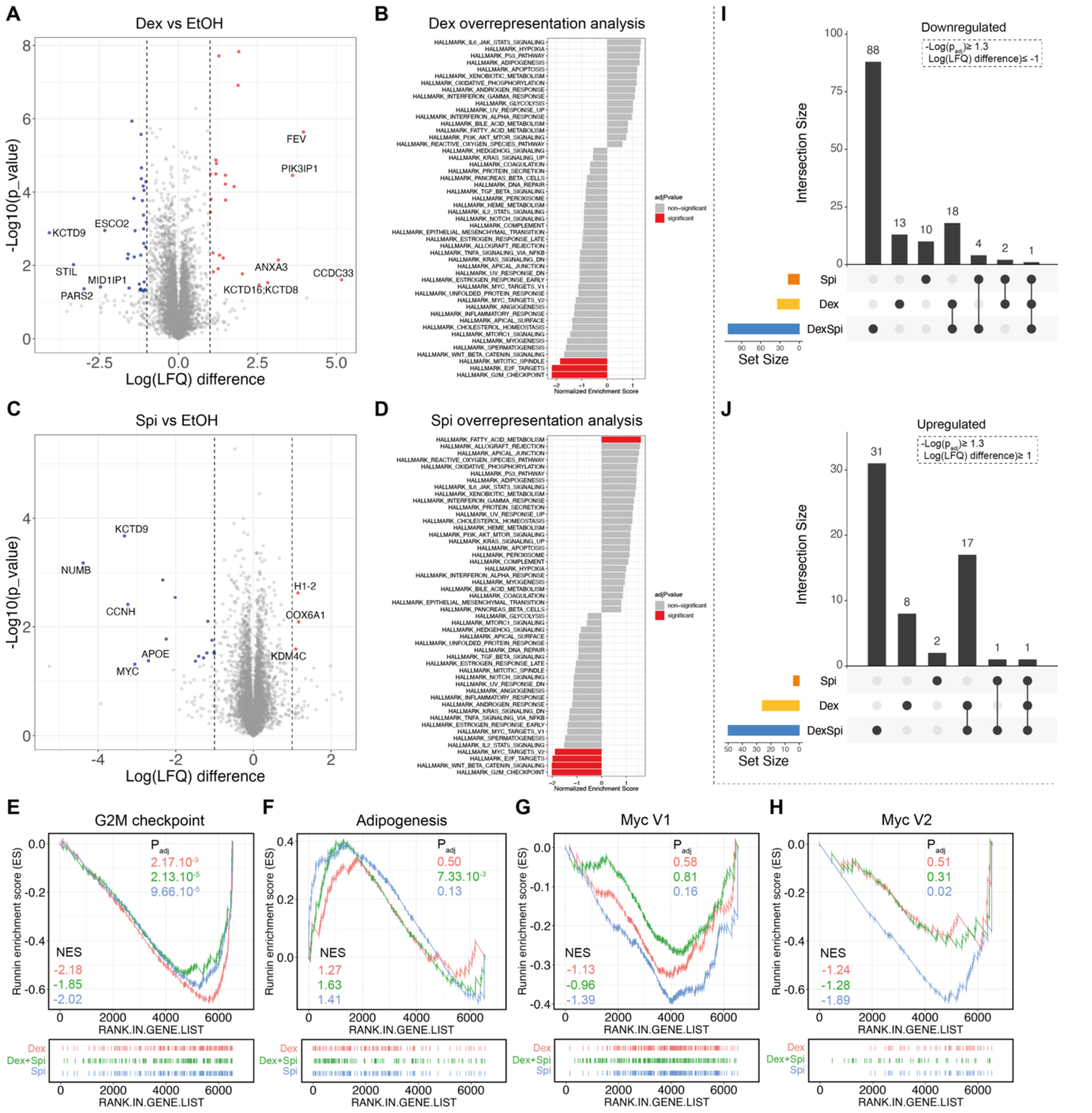

Fig. S8: MS-based shotgun proteomics of other pairwise comparisons along with additional single hallmark plots enriched upon Dex-Spi treatment.

(**A, C**) Volcano plots depicting the p_{adj} (log10 scale) in function of the log(LFQ) in the pairwise comparison (**A**) Dex vs EtOH and (**C**) Spi vs EtOH. Significantly regulated proteins -log(padj)≥1.3 are colored in red (log(LFQ)>1, upregulated) or blue (log(LFQ)<-1, downregulated); non-significant proteins (-log(p_{adj})<1.3) in grey.

(**B, D**) GSEA-based overrepresentation analysis for the proteins regulated by (**B**) Dex or (**D**) Spi, hereby identifying hallmarks that are significantly (red) or non-significantly (grey) enriched.

(**E-H**) GSEA of single hallmarks, i.e. (**E**) G2M checkpoint (**F**) Adipogenesis, (**G**) Myc V1 (**H**) Myc V2 targets, for each pairwise comparison, along with the respective normalized enrichment score (NES) and p_{adj}.

(**I-J**) Upset plots depicting the number of proteins that are (**I**) downregulated or (**J**) upregulated in and between pairwise comparisons (Dex-Spi vs EtOH, Dex vs EtOH and Spi vs EtOH).

Figure S9

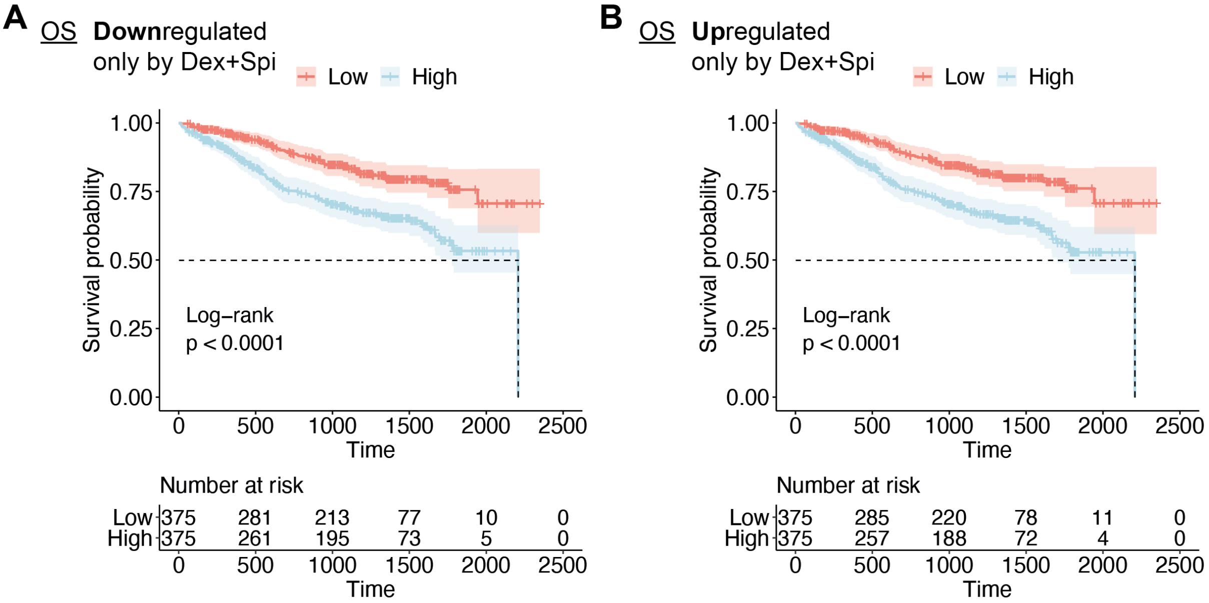

Fig. S9: OS analysis of the CoMMpass cohort using differentially regulated proteins as input. (A-B) Kaplan-Meier curve of the CoMMpass patient cohort (N=750), depicting the survival probability in function of overall survival (OS) for low or high expression of proteins that were uniquely (**A**) downregulated or (**B**) upregulated by the Dex-Spi combination. Statistical analyses were performed in R (package survival), using a log-rank test.

Table S1. siRNA target sequence identifiers.

Gene (protein)	siRNA information
NR3C1 (GR)	M-003424-03-0010, siGENOME Human NR3C1 siRNA – SMARTpool (mixture of 4 siRNA's):<br>• siGENOME SMARTpool siRNA D-003424-04 NR3C1<br>• siGENOME SMARTpool siRNA D-003424-06 NR3C1<br>• siGENOME SMARTpool siRNA D-003424-19 NR3C1<br>• siGENOME SMARTpool siRNA D-003424-20 NR3C1
NR3C2 (MR)	M-003425-02-0010, siGENOME Human NR3C2 siRNA – SMARTpool (mixture of 4 siRNA's):<br>• siGENOME SMARTpool siRNA D-003425-01 NR3C2<br>• siGENOME SMARTpool siRNA D-003425-02 NR3C2<br>• siGENOME SMARTpool siRNA D-003425-04 NR3C2<br>• siGENOME SMARTpool siRNA D-003425-05 NR3C2
Non-targeting (Control)	D-001206-13-20, siGENOME Non-targeting siRNA Pool 1

Table S2. Sequences of qPCR primers.

Target	Primer forward	Primer reverse
MR	CAGGGGATGCACCAAATCAG	AGGCCATCCTTTGGAATTGTG
GR	TGATGAAGCTTCAGGATGTCA	TTCGAGCTTCCAGGTTCATTC
FKBP5	AGTAGAAATCCACCTGGAAGGC	ATTTAGGCTTCCCTGCCTCT
TSC22D3	GCGTGAGAACACCCTGTTGA	TCAGACAGGACTGGAACTTCTCC
SGK1	GAGATTGTGTTAGCTCCAAAGC	CTGTGATCAGGCATACCACACT
TMEM119	GACCCCTGCACACATTACGA	TGTTTCCGTAGAGTGCCTCG

Table S3. Primary antibodies.

Target	Catalog number	Obtained from
MR 6G1	NA	Dr. Gomez-Sanchez (Univ. of Mississippi)
GR H300	sc-8992	Santa Cruz Biotechnology
GR G5	sc-393232	Santa Cruz Biotechnology
PARP	556494	BD Biosciences
Cleaved-caspase 3	9664	Cell Signaling
Bim	sc-374358	Santa Cruz Biotechnology
Bcl-XL	sc-8392	Santa Cruz Biotechnology
β-catenin	C7202	Sigma
LC-3	L8918	Sigma
Hsp70	ADI-SPA-810	Enzo life sciences
RNA pol2	sc899	Santa Cruz Biotechnology
RNA pol2 (P-Ser2)	ab5095	Abcam
c-myc	CST5605T	Cell signaling
HEXIM	CST12604S	Cell signaling
Cyclin-D1	CST5506S	Cell signaling
CCL3	ab259372	Abcam
REDD1	10638-1-AP	Protein tech
IRE1α	sc-390960	Santa Cruz Biotechnology
BOB-1	CST43079	Cell signaling
GAPDH	ab9485	Abcam
GAPDH	G8795	Sigma
Tubulin	T5168	Sigma

Table S4. Comparison of *NR3C2* mRNA levels between MM cell lines. The Keats lab (https://www.keatslab.org/) has built a transcriptomic resource of MM cell lines by subjecting these to RNA-sequencing. The fragments per kilobase per million (FPKM) values for *NR3C2* (MR) were extracted for the cell lines used in this study. In addition, per cell line the Cq values of *NR3C2* of all biological replicates of the solvent condition (EtOH, in Fig.1A QPCR) were averaged and the SD was determined.

	Keats_lab FPKM	QPCR_EtOH Cq_mean	QPCR_EtOH Cq_SD
MM1.S	1.22478	27.16	0.63
OPM-2	3.05642	25.04	0.24
U-266	0.0712736	32.44	1.64
L-363	0.347215	31.25	0.46
MM1.R	0.940151	26.84	0.41

Table S5. RNA-seq-derived interaction term genes. The baseMean, log2FC and p_{adj} of these genes are mentioned.

**Gene**	**baseMean**	**Log2FC**	p_{adj}
STK38L	4064.5	0.57	1.46E-09
TFRC	2334.4	0.46	4.80E-02
CCR1	3376.0	0.38	3.10E-03
SCD	13024.2	0.38	4.80E-02
BARX2	2207.5	0.34	1.86E-02
JCHAIN	83330.9	0.34	1.25E-02
CD28	6686.1	0.32	2.80E-02
POU2AF1	9033.7	0.31	3.51E-02
PLXNC1	7038.2	0.31	1.46E-03
METTL7A	16737.2	0.25	4.69E-02
PM20D2	3878.5	0.23	2.85E-02
PFKFB2	5155.7	-0.25	4.86E-02
ERN1	9782.2	-0.27	3.45E-02
GLUL	5023.7	-0.31	2.85E-02
TREML2	3849.4	-0.31	8.37E-03
SLFN5	1586.6	-0.31	3.90E-02
SMC4	22296.2	-0.32	4.54E-03
BACH2	1608.1	-0.35	3.22E-02
CELF2	12208.6	-0.36	1.04E-02
CYSLTR1	2308.5	-0.37	2.44E-02
PLEKHA7	3633.0	-0.37	7.16E-03
IRAG2	2114.1	-0.38	1.16E-02
FKBP5	9001.7	-0.42	1.46E-03
GCSAM	2554.6	-0.44	8.31E-03
SESN1	3963.5	-0.44	3.10E-03
SLC38A2	6626.6	-0.45	4.54E-03
MEI1	2981.3	-0.46	9.73E-03
INSR	2944.2	-0.46	5.56E-05
HMGB3	2430.2	-0.50	2.23E-06
TMSB4X	16052.7	-0.52	3.66E-05
ZC3H12A	3422.8	-0.73	1.08E-08
NUDT16	2981.9	-0.79	8.62E-17
RHOB	1914.3	-0.82	1.20E-04
TSC22D3	12654.5	-0.82	3.45E-02
TXNIP	19898.5	-1.02	1.08E-09
FBXO32	2535.1	-1.10	5.12E-14
DDIT4	3500.6	-1.67	5.24E-07
